# FDA-Approved Janus Kinase-Signal Transducer and Activator of Transcription (JAK-STAT) Inhibitors for Managing Rheumatoid Arthritis: A Narrative Review of the Literature

**DOI:** 10.7759/cureus.59978

**Published:** 2024-05-09

**Authors:** Tejaswini Potlabathini, Mounica A Pothacamuri, Venkata Varshitha Bandi, Mahnoor Anjum, Parmendra Shah, M. Molina, Nilashis Dutta, Oleksandr Adzhymuratov, Midhun Mathew, Vatsalya Sadu, Shiza A Zahid, Harini Lingamgunta, Monika Sahotra, Syed Muhammad Zain Jamil Nasiri, Christine Dawn M Daguipa

**Affiliations:** 1 Internal Medicine, Malla Reddy Institute of Medical Sciences, Hyderabad, IND; 2 Internal Medicine, Kasturba Medical College, Mangalore, Mangalore, IND; 3 Medicine and Surgery, Guntur Medical College, Guntur, IND; 4 Medicine, King Edward Medical University, Lahore, PAK; 5 Internal Medicine, Dali University, Dali, CHN; 6 Internal Medicine, International Medical Graduates (IMG) Helping Hands, Newark, USA; 7 General Medicine, North Bengal Medical College and Hospital, Siliguri, IND; 8 Medicine and Surgery, Dnipro State Medical University, Dnipro, UKR; 9 Internal Medicine, Pennsylvania Hospital, Philadelphia, USA; 10 Medicine and Surgery, Kamineni Academy of Medical Sciences and Research Centre, Hyderabad, IND; 11 Medicine and Surgery, Jinnah Sindh Medical University, Karachi, PAK; 12 Medicine, All Saints University School of Medicine Dominica, Chicago, USA; 13 Internal Medicine, International Medical Graduates (IMG) Helping Hands, San Pablo, USA; 14 Anesthesia and Critical Care, Ibn-e-Siena Hospital & Research Institute, Multan, PAK; 15 Medicine, Far Eastern University - Dr. Nicanor Reyes Medical Foundation, Quezon City, PHL

**Keywords:** clinical efficacy, cost effective, fda-approved medications, teratogenic, rheumatoid arthriitis, janus kinase (jak) inhibitors

## Abstract

Rheumatoid arthritis (RA) is a complex autoimmune disease causing chronic joint inflammation and, in more serious cases, organ involvement. RA typically affects people between the ages of 35 and 60; however, it can also afflict children younger than the age of 16 years and can also demonstrate a pattern of remission later in the disease course. Non-steroidal anti-inflammatory drugs, glucocorticoids, exercise, and patient education are all used in the management of RA, which is divided into symptomatic management and disease-modifying management (disease-modifying antirheumatic drugs) to reduce pain and inflammation, thereby preserving joint function. Janus kinase inhibitors (JAKis) have led to a substantial improvement in the management of RA. By specifically targeting the JAK-signal transducer and activator of transcription pathway, which is essential for immunological modulation, these inhibitors also demonstrate promise in treating various autoimmune illnesses, including inflammatory bowel diseases, giant cell arteritis, ankylosing spondylitis, and psoriatic arthritis. Tofacitinib, baricitinib, upadacitinib, peficitinib, delgocitinib, and filgotinib are examples of FDA-approved JAKis that have distinct properties and indications for treating a range of autoimmune illnesses. JAKis demonstrate a promising treatment approach for managing RA and other autoimmune diseases while enhancing patient outcomes and quality of life. However, due to major safety concerns and the need for long-term success, meticulous patient monitoring is essential.

## Introduction and background

Clinical background of rheumatoid arthritis (RA)

RA, a chronic autoimmune disease, is characterized by symmetrical inflammation. While largely affecting small joints, RA can also involve larger joints, such as the skin, eyes, heart, kidneys, and lungs. Due to cartilage and bone destruction, RA frequently causes painful symptoms and joint abnormalities [1]. Rheumatoid nodules under the skin, tiredness, fever, weight loss, sore and swollen joints, warmth in the affected areas, and morning stiffness lasting more than 30 minutes are common symptoms of RA. RA normally presents between 35 and 60 years of age, marked by remission and phases of exacerbation. Juvenile RA, which resembles polyarticular RA but lacks the presence of rheumatoid factor, can also affect people under 16 years of age [2-5]. While RA affects roughly 1% of the population in the United States [6], it is thought to be prevalent in Western populations at a rate of 1-2% [5,7]. Unlike RA, which predominantly manifests due to autoimmune processes rather than wear and tear, osteoarthritis (OA) does not affect the lungs, heart, or immune system. In addition, as opposed to RA’s symmetrical pattern, OA typically has an asymmetrical presentation. Another important distinction is the prolonged presence of morning stiffness. While morning stiffness normally lasts 20-30 minutes in patients with OA, it lasts at least an hour in patients with RA [8,9].

Management of RA

Reducing joint pain and inflammation, enhancing joint mobility, and protecting against joint degradation are the main goals of treatment for RA. Comprehensive treatment plans include pharmaceutical interventions, weight-bearing exercises, patient education, and rest. These therapies are tailored to each patient’s needs, taking into account several variables, including disease progression, joint involvement, age, general health, occupation, adherence to therapy, and patient preference [10]. Current RA treatment options concentrate on two main aspects, namely, symptomatic management, which involves the use of non-steroidal anti-inflammatory drugs (NSAIDs) and glucocorticoids (GCs), and disease-modifying management (disease-modifying antirheumatic drugs, DMARDs), and secondly, following recommendations from the American College of Rheumatology (ACR) and the European League Against Rheumatism (EULAR) [11,12]. NSAIDs and GCs are used in periods of discomfort; patients should not be on steroids or NSAIDs on a regular basis. Under certain circumstances, the use of mild opioid analgesics for temporary pain relief depends on a careful analysis of the benefit-to-risk ratio [13,14].

DMARDs are intended to elicit remission by suppressing autoimmune activity and delaying or preventing joint deterioration. Certain DMARDs can take six weeks to six months to illustrate the effect. Hence, starting treatment promptly is crucial. Conventional synthetic DMARDs (csDMARDs), biologic DMARDs (bDMARDs), and targeted synthetic DMARDs (tsDMARDs) are the three types of DMARDs [15]. CsDMARDs are recommended, as per ACR and EULAR 2022, as the initial course of treatment for patients with newly diagnosed RA. The use of bDMARDs or tsDMARDs, particularly Janus kinase inhibitors (JAKis), may be considered if the first-line therapy proves unsuccessful. The oral administration route of tsDMARDs, including JAKis, is one of their major benefits [16]. Secondly, having rapid action as compared to the other DMARDs makes them an excellent option.

## Review

Recently approved JAKis for RA

Upadacitinib is a recently FDA-approved second-line medication for patients with moderate to severe active RA with an inadequate response to methotrexate (MTX) or who have developed intolerance [17]. This medication is a second-generation selective JAKi, predominantly targeting the JAK1 enzyme [18]. On August 16, 2019, the FDA authorized upadacitinib based on promising results from phase III studies globally that included patients with moderate to severe RA [19]. It is crucial to emphasize that combining upadacitinib with other JAKis or potent immunosuppressive medications such as azathioprine and cyclosporine is not recommended. Simultaneously, its use is discouraged with biological DMARDs. Notably, when combined with MTX, the first-line medication, upadacitinib has been demonstrated to reduce disease progression as determined by radiographic imaging while maintaining therapeutic efficacy [17]. Current clinical research examines the use of comparable medications to treat additional autoimmune diseases. These conditions include psoriatic arthritis (PA), atopic dermatitis (AD), ankylosing spondylitis (AS), giant cell arteritis, and inflammatory bowel disorders (IBDs), such as Crohn’s disease and ulcerative colitis (UC) [19], with different JAK-signal transducer and activator of transcription (STAT) inhibitors being approved for each IBD.

JAK-STAT inhibitors

Background

Intracellular, non-receptor tyrosine kinases are known as JAKs [20]. JAK1, JAK2, JAK3, and TYK2 are the four members of the JAK family. Many efforts have been undertaken to comprehend the structure and functions of JAKs since their discovery 30 years ago [21]. The four JAKs are crucial for the JAK-STAT pathway, which is involved in transmitting cytokine-mediated signals [22]. Four JAK family members have been identified as therapeutic targets for various illnesses [23].

Mechanism of Action of JAK-STAT

It has been noted that several ligands, including cytokines and growth factors, activate the JAK-STAT pathway [24]. The phosphorylation and dimerization of STATs follow the activation of JAKs. The phosphorylated STATs move into the nucleus and start the transcriptional response in the genes that regulate hematopoiesis, inflammation, and immunity [24-26].

Clinical Use of JAK Inhibition

The JAK-STAT pathway transmits growth factors and cytokines, which are essential in autoimmune disorders and inflammation [27]. JAK1 appears to be one of these kinases that is particularly important in pruritic dermatitis, allergic rhinitis, asthma, and inflammatory bowel disease [28-31]. Small-molecule JAKis have demonstrated efficacy in treating the aforementioned disorders [30,32,33]. RA, psoriasis, and pruritis have all been successfully treated with several small compounds with JAK1 and JAK2 inhibitory activities [34]. Additionally, the effectiveness of several JAK3 selective inhibitors in the treatment of RA has been extensively studied [35]. Dual JAK1/TYK2 inhibitors have also been investigated as potential treatments for inflammatory disorders [34,36].

However, increased JAK activation has also been associated with many cancer types [37]. Solid tumor angiogenesis and proliferation are both significantly influenced by the JAK/STAT3 pathway [37]. JAK2 mutation (JAK2V617F) in myeloproliferative neoplasms was discovered in 2005 and has received much attention [38,39]. Our understanding of this mechanism has improved our knowledge of these illnesses. JAK2V617F has also been identified as a potential therapeutic target for myeloproliferative neoplasms [40,41]. Ruxolitinib, a JAK1/JAK2 inhibitor, has been approved for treating polycythemia vera and myelofibrosis [42,43]. Additionally, the concurrent inhibition of JAK2 and FLT3 may present further therapeutic options for treating acute myelogenous leukemia and myeloproliferative neoplasms [44-46].

FDA-approved JAKis for clinical use

Tofacitinib

Tofacitinib was given FDA approval in 2012 to treat RA [47].

Baricitinib

The FDA authorized baricitinib to treat RA in 2017 [48].

Fedratinib, Upadacitinib, and Peficitinib

Three JAKis, namely, fedratinib, upadacitinib, and peficitinib, were authorized for clinical use in 2019 for RA patients by the FDA. Peficitinib had already received approval in Japan for the management of RA earlier [19,49,50].

Delgocitinib and Filgotinib

In 2020, filgotinib and delgocitinib received licenses in Japan to treat AD and RA, respectively [51,52].

Baricitinib

It is an orally active small-molecule JAK1/2 inhibitor. It was approved in 2017 by the European Medicines Agency (EMA) to treat RA [48]. To treat moderate to severe RA, the FDA approved baricitinib in June 2018 [47]. Recently, the FDA authorized using baricitinib for treating COVID-19 in hospitalized patients as well [53].

Filgotinib

It is classified as an adenosine triphosphate-competitive JAK1-specific inhibitor [52]. In September 2020, the EMA authorized filgotinib for use in adults with moderately to severely active RA [52]. Filgotinib was also approved in Japan to treat RA [52]. The approval of filgotinib for treating RA was prompted by efficacious clinical outcomes, which demonstrated that RA may be managed with a particular JAK1 inhibitor [54]. Later, two filgotinib randomized phase IIa studies were carried out by Vanhoutte et al., which provided evidence that the medication would be useful in treating RA [55]. Additionally, filgotinib demonstrated a rapid improvement in RA symptoms [56]. For the treatment of moderate to severe RA, filgotinib received approval in 2020. It functions as a JAK1-selective inhibitor to prevent the activation and phosphorylation of STAT [22]. Similar assessments of filgotinib’s safety and efficacy for treating active PA were made in a clinical study (NCT03101670) [57].

Peficitinib

Japan approved its use for treating RA in 2019 [50]. In an experimental study, peficitinib decreased bone loss and paw swelling in rats with adjuvant-induced arthritis [58]. In a clinical study (NCT01565655), peficitinib exhibited a dose-dependent ACR20 response rate when given orally to patients with moderate to severe RA [59]. Additionally, in Asian patients who did not react well to conventional DMARDs, peficitinib demonstrated clinical effectiveness and prevented joint deterioration [60]. Peficitinib’s effectiveness also held true after a lengthy course of therapy [61].

Tofacitinib

Tofacitinib, a JAKi, was licensed by the FDA in 2012 to treat RA [47,62,63]. It was also approved for treating PA and UC in 2017 and 2018, respectively [64]. Tofacitinib was also given FDA approval in 2020 to treat juvenile idiopathic arthritis [65]. In December 2021, tofacitinib was approved for treating active AS [66]. Several clinical trials have closely monitored the effectiveness of tofacitinib in treating RA. Data from a clinical trial (NCT00814307) in 2008 studying the clinical efficacy of tofacitinib for RA revealed the resolution of RA signs and symptoms upon administration [67]. In a separate clinical study (NCT00853385), tofacitinib showed equivalent effectiveness to adalimumab in patients with RA [68]. Additionally, in individuals receiving MTX, tofacitinib stopped the course of structural deterioration [69]. Numerous studies have examined the efficacy of tofacitinib in treating a range of inflammatory and immunological conditions since its initial authorization in 2012 for treating RA. This can be evidenced by a clinical study (NCT01882439), where tofacitinib reduced active PA in those who did not react well to tumor necrosis factor inhibitors [70].

Mease et al. studied tofacitinib’s efficacy in treating PA patients who did not react well to DMARDs [71]. Another clinical study (NCT00787202) examined the clinical efficacy and safety of tofacitinib to treat patients with severely active UC [72]. The findings showed that patients receiving tofacitinib were more likely to experience a favorable clinical outcome and remission than individuals receiving a placebo. Huang et al. also discovered that the arthritis of a 13-year-old female improved with complete remission in just three months of starting the treatment [73]. The FDA has also approved tofacitinib to treat active PA, UC, and juvenile idiopathic arthritis [62,65].

Upadacitinib

Upadacitinib was recently approved to treat PA [74], based on its favorable potency in treating RA as per recent clinical studies [17]. Additionally, Smolen et al.’s assessment of the efficiency of upadacitinib monotherapy in treating RA demonstrated significant therapeutic results when compared to MTX [75]. Upadacitinib’s usage, alone or in combination with other medications, has been associated with lower direct medical costs [76]. In August 2019, upadacitinib was approved to treat moderate to severe RA. Many clinical trials were also carried out to evaluate the efficiency of upadacitinib in the management of PA. In a clinical study for treating PA (NCT03104400), upadacitinib dramatically improved patient outcomes [77].

Upadacitinib was evaluated through a 24-week phase III clinical study to treat PA [78]. The results illustrated that 30 mg of upadacitinib given daily performed better than the group receiving adalimumab. Additionally, the trial of upadacitinib for PA patients did not reveal any major safety concerns. Burmester et al. also evaluated the safety of upadacitinib in patients with PA for up to three years, and the results revealed a safety profile similar to that of RA [79]. Upadacitinib has been given current [74,80] FDA and EMA approval for treating people with active PA.

When used with MTX, the first-line medication upadacitinib slowed the course of the illness on radiographic imaging and retained therapeutic effectiveness [17]. Upadacitinib causes several upper respiratory tract infections [81]. For monotherapy, a daily oral dose of 15 mg is advised [19]. Taking a single 15-mg tablet whole without breaking or crushing it when taking the prescription is recommended, which can be taken with or without food. In placebo-controlled studies, the adverse effects were observed when individuals took 15 mg of upadacitinib orally [19]. In a phase III double-blind, randomized, controlled clinical trial, more severe side effects, such as herpes zoster virus and serious infections, were observed in participants given 30 mg (1%) [81]. Additionally, GI perforations, thrombosis, and cancer have all been linked in clinical research to the concurrent use of NSAIDs [19].

## Conclusions

A multimodal strategy is required to manage RA, including medications, physical activity, patient education, and rest. The availability of disease-modifying drugs such as JAKis, critical in controlling autoimmune activity and avoiding joint degradation, has considerably uplifted the therapeutic options. Upadacitinib, a JAKi, has been approved by the FDA as a second-line treatment for moderate to severe RA, especially for patients who do not respond well to MTX. Upadacitinib targets the JAK-STAT pathway, which plays a crucial role in immunological and inflammatory responses and is a valuable therapeutic target for various autoimmune diseases other than RA. The approval of numerous JAKis, including tofacitinib, baricitinib, filgotinib, and peficitinib, underscores the significance of this approach in treating numerous autoimmune diseases. The effectiveness of these medications has been shown in treating RA and PA, UC, and juvenile idiopathic arthritis. JAKis’ potential uses in various autoimmune disorders and even some malignancies are being investigated as research progresses, promising better treatment outcomes and quality of life. Although these medications show promise, it is crucial to consider their safety profiles and possible side effects in clinical practice when making treatment decisions to improve patient care.
